# Case Report: A Patient With COVID-19 Who Benefited From Hemoadsorption

**DOI:** 10.3389/fmed.2020.607849

**Published:** 2020-12-01

**Authors:** Yinke Du, Zhipeng Qi, Jiangwei Ma, Da Sun, Li Yao, Bin Xu, Wei Liu, Zhaofa Xu, Yu Deng

**Affiliations:** ^1^Department of Nephrology, First Hospital of China Medical University, Shenyang, China; ^2^Department of Environmental Health, School of Public Health, China Medical University, Shenyang, China; ^3^Department of Pulmonary and Critical Care Medicine, First Hospital of China Medical University, Shenyang, China

**Keywords:** 2019-nCoV, COVID-19, epidemic, extracorporeal blood purification, treatment

## Abstract

In December 2019, the 2019 novel coronavirus disease (COVID-19), which has been identified to be caused by severe acute respiratory syndrome coronavirus 2 (SARS-CoV-2), emerged in China and spread across the world. Higher plasma levels of cytokines, including interleukin (IL)-6, IL-2, IL-7, IL-10, and tumor necrosis factor-α, were found in patients with COVID-19, which implies the occurrence of a cytokine storm and its association with disease severity. Extracorporeal blood purification has been proven to effectively remove the released inflammatory cytokines. In this study, we report on a patient with COVID-19 who benefited from hemoadsorption.

## Introduction

A novel coronavirus, coronavirus 2019 (COVID-19), emerged in Wuhan, Hubei, China, in December 2019, quickly posing a significant threat to global health. At the time this article was written, the novel coronavirus had affected more than 211 countries and regions, resulting in more than 5.5 million infections and more than 340,000 deaths worldwide. According to a new report, the mortality rate for critical cases has been recorded as high as 60.5% (1). At present, the newly identified novel coronavirus mostly affects the respiratory system, ranging from mild flu-like symptoms to hypoxic respiratory failure, acute respiratory disease syndrome (ARDS), multi-organ failure, shock, and even death. Unfortunately, the pathogenesis of COVID-19 remains unclear, and there are no efficient therapeutics.

Wang et al. have reported that cytokine storms are involved in the pathogenesis of COVID-19 (2). A cytokine storm involving a considerable release of proinflammatory cytokines occurred, including interleukin (IL)-6, IL-2, IL-7, IL-10, and tumor necrosis factor (TNF)-α. This implied the occurrence of a cytokine storm and its association with disease severity (3). The cytokine storm may result in increased alveolar-capillary blood-gas exchange dysfunction, particularly impaired oxygen diffusion, and eventually lead to pulmonary fibrosis and organ failure (4). Thus, we hypothesized that eliminating the cytokine storm in the early stage could mitigate the injury to the lungs and the body. Hemoadsorption has been identified as a novel extracorporeal blood purification therapy aimed at a non-selective reduction of the circulating levels and activities of both pro- and anti-inflammatory mediators. In this study, we report on a case of a COVID-19 patient with cytokine storm, who have fully recovered by extracorporeal blood purification.

## Case Report

**Figure 1:**
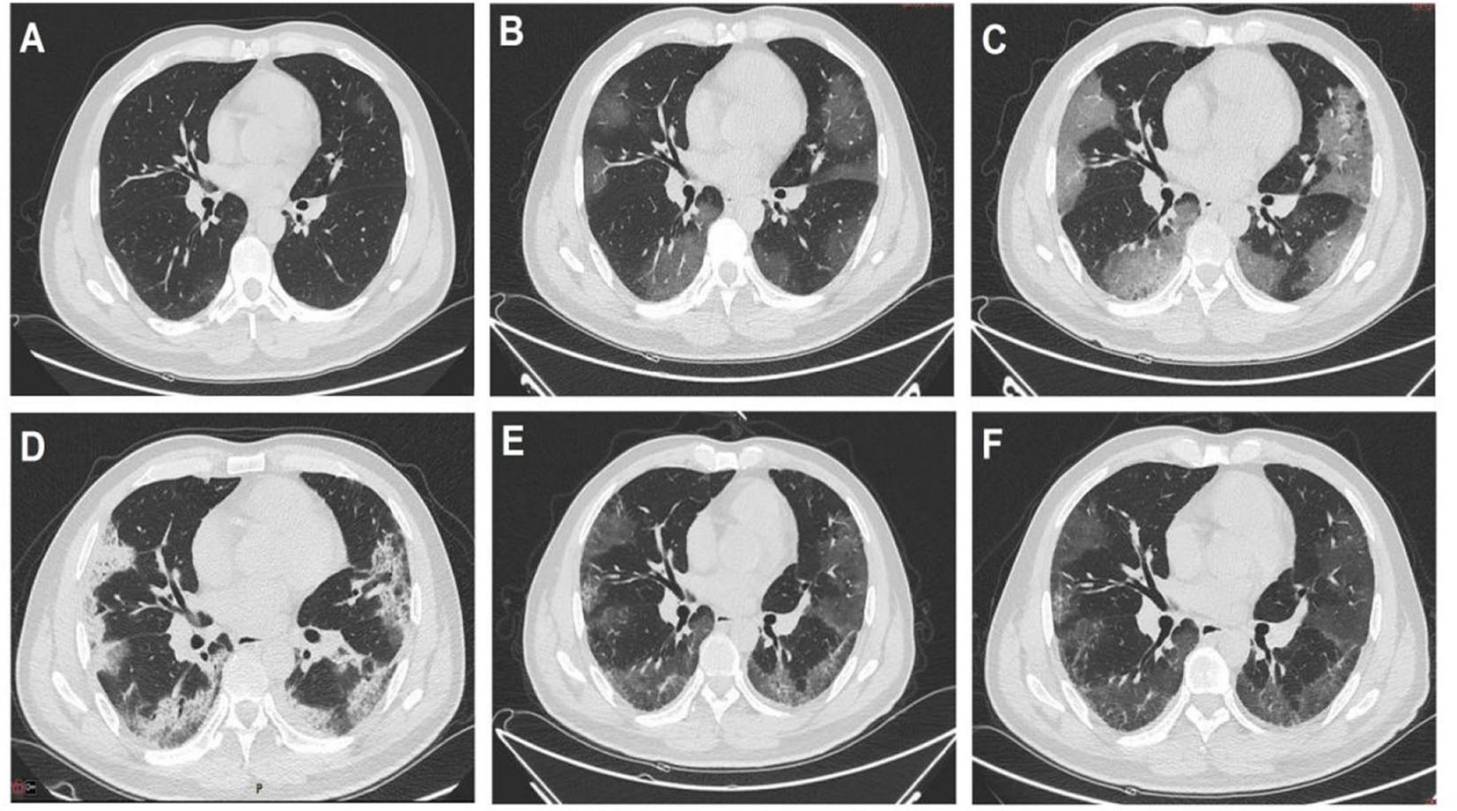
Dynamic change shown on the chest computed tomography manifestations of the patient. **(A)** On January 30, only small ground-glass opacity (GGO) was seen. **(B)** On February 4, the GGO had enlarged in both lungs and was peripherally distributed. **(C)** On February 9, the infiltration had increased further. **(D)** On February 17, the lesion was consolidated and fibrosis occurred. **(E)** On March 1, the lesion absorbed significantly. **(F)** On March 10, only a small area of GGO and fibrosis remained.

A 46-year-old man was admitted to our department on February 10, 2020, with complaints of cough, fever for 10 days and shortness of breath for 5 days. He developed cough and fever on January 31, 2020 (day 0), and short of breath on day 4 (February 4, 2020). Chest computed tomography (CT) showed bilateral ground-glass opacity (GGO), with peripheral distribution which progressed rapidly (Figures 1A,B). The COVID-19 nuclear acid polymerase chain reaction test was positive and arbidol was prescribed on day 4 (Table 1). On admission (day 10), the patient still suffered fever with body temperature 38.9°C. The initial laboratory findings have been listed in Table 2. Arterial blood gas showed PaO_2_/FiO_2_ was 294 mmHg and oxygen (3 L/min) via a nasal catheter was started. On day 14 (February 14, 2020), his fever persisted, so we added ribavirin and lopinavir/ritonavir (Table 1). On day 17, the patient's dyspnea became worse, PaO_2_/FiO_2_ decreased to 245 mmHg. The nasal cannula oxygen inhalation was switched to mask oxygen (8 L/min), whereas PaO_2_/FiO_2_ decreased to 207 mmHg. Chest CT showed multiple “paving stone” -like density shadows under the pleura, interlobular septum, and the pleura thickening (Figures 1C,D). On day 18, laboratory examinations showed that multiple indicators had increased, particularly IL-6, IL-8, and TNF-α (Table 2). On day 19, the patient had no fever but was still struggling to breathe. We then changed to high-flow nasal cannula oxygen therapy (30 L/min; FiO_2_ 43%); the PaO_2_/FiO_2_ decreased to 176 mmHg; and he was given methylprednisolone 40 mg bid intravenously (Table 1). On day 20, his PaO_2_/FiO_2_ decreased to 167 mmHg, on day 23, the laboratory test showed a sudden increase in multiple indicators (Table 2).

**Table 1:** The details of therapy strategies.

**Therapy**	**Jan 31**	**Feb 13**	**Feb 14**	**Feb 18**	**Feb 19**	**Feb 20**	**Feb 21**	**Feb 23**	**Feb 25**	**Feb 27**	**Mar 23**
Nasal catheter oxygen											
High flow oxygen											
Moxifloxacin (400 mg qd po)											
Arbidol (0.2 g tid po)											
Ribavirin (500 mg qd ivgtt)											
Lopinavir/ritonavir (400 mg/100 mg bid po)											
Human immunoglobulin (20 g qd ivgtt)											
Methylprednisone (40 mg bid iv)											
Hemofiltration and perfusion (hemofiltration 8 h, hemoperfusion 2.5 h)											

*Different colors represent different treatment strategies*.

**Table 2:** The results of laboratory investigations.

**Indicators**	**Feb 11**	**Feb 14**	**Feb 18**	**Feb 23**	**Feb 25**	**Feb 29**	**Mar 3**	**Mar 10**
WBC × 10^9^/L	7.09	6.38	8.5	14.5	15.9	13.7	7.23	4.89
Neutrophil (%)	82.8	74	78.2	88.8	89.6	88.1	72.6	67.5
CRP mg/L	59	–	44	32	27	28	23	18
PCT ng/ml	0.06	0.08	0.06	2.4	2.2	1.8	0.72	0.12
IL-6 pg/ml	1.8	1.4	5.8	43.5	29.1	6.1	5.6	2.3
IL-8 pg/ml	<5	<5	7.5	78.5	25.3	11.9	5.8	6.2
TNF-α pg/ml	3.2	–	10.2	67.9	60.5	21.7	11.7	4.7
AST U/L	217	–	117	34	33	35	–	36
ALT U/L	154	–	78	40	39	38	–	41
Scr umol/L	65	–	64	71	65	57	–	63
PaO_2_/FiO_2_	294	288	245	217	307	368	–	361

**Figure 2:**
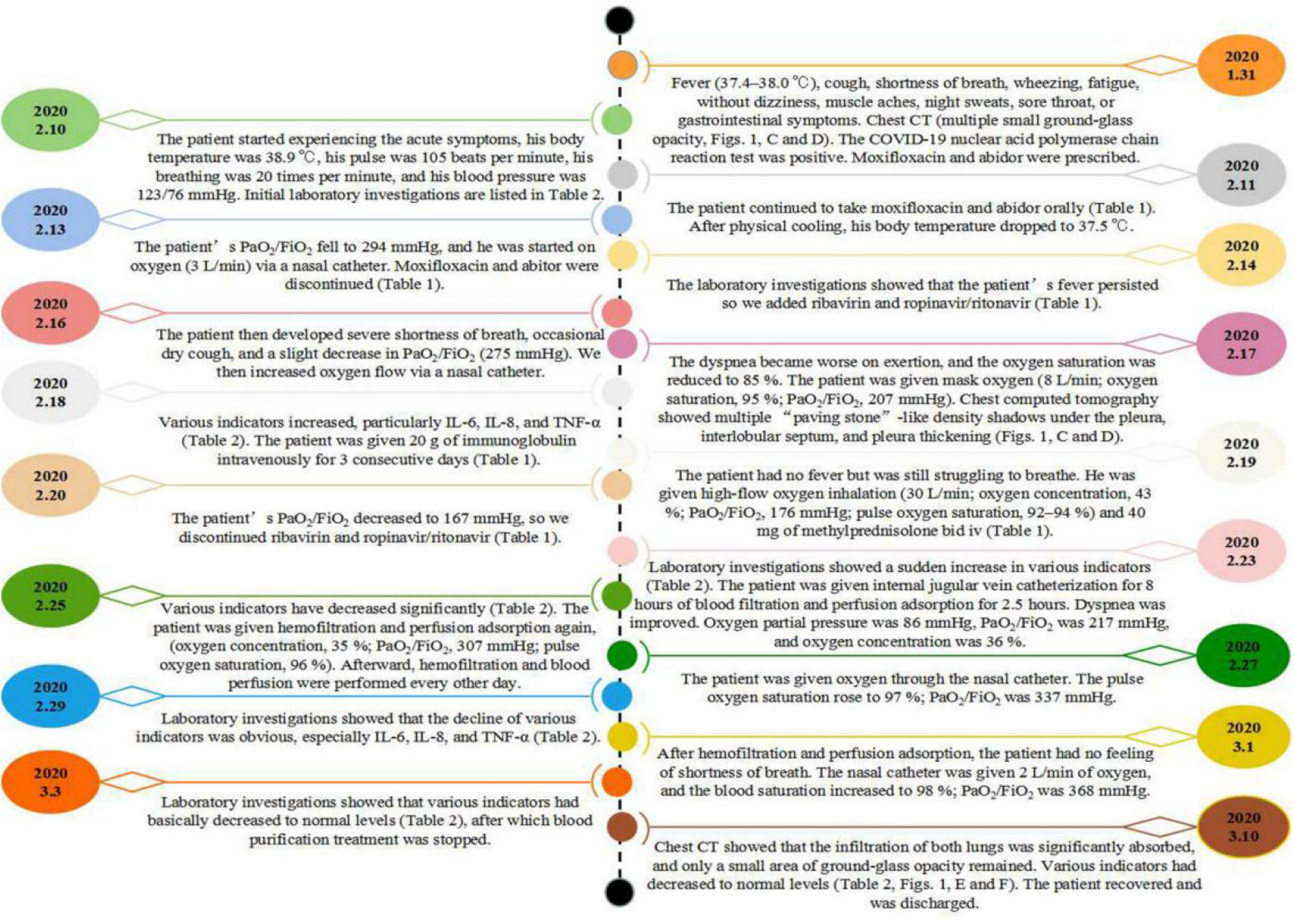
Timeline of the reported case.

Considering that the patient had an inflammatory factor storm, the PaO_2_/FiO_2_ was worse than before. The patient was given internal jugular vein catheterization for 8 h of blood filtration and 2.5 h of perfusion adsorption on day 23 (February 23, Table 1). The reexamination of blood-gas results showed that the PaO_2_/FiO_2_ was 217 mmHg. On day 25 (February 25), the indicators were significantly decreased (Table 2). The patient was then given hemofiltration and perfusion adsorption again. After treatment, the PaO_2_/FiO_2_ increased to 307 mmHg. Afterward, hemofiltration and blood perfusion were performed every other day. On day 27 (February 27), the oxygen inhalation was switched to nasal cannula. The PaO_2_/FiO_2_ increased to 337 mmHg. On day 29 (February 29), laboratory test showed that the decline of indicators was obvious, especially IL-6, IL-8, and TNF-α (Table 2). On day 31 (March 1), after hemofiltration and perfusion adsorption, the patient demonstrated no shortness of breath. The PaO_2_/FiO_2_ was 368 mmHg. On March 3, the laboratory results showed that various indicators had basically decreased and reached normal levels (Table 2), blood purification treatment was stopped. Chest CT on March 10 showed that the infiltration of both lungs was significantly absorbed, and only a small ground-glass opacity was left. The laboratory test showed that indicators had decreased to normal levels (Table 2, Figures 1E,F). The patient recovered and was later discharged from the hospital (the intuitive treatment process can be seen in Figure 2).

## Discussion

In the absence of effective antiviral treatments, respiratory, and circulation support were almost the last defense for severe COVID-19 with ARDS (5). In COVID-19, the median time from first symptom to dyspnea was 5 days, and to ARDS was 8 days (6). Several studies have reported that cytokine storm has been disclosed as a main pathological characteristic of SARS-CoV-2 (7, 8). In addition, Wang et al. (2) have reported that a cytokine storm plays a major role in COVID-19 and it is also the direct pathogenic contributor to induce ARDS. Therefore, in severe disease, extracorporeal treatment options seem to be a good approach (9).

Hemoadsorption was suggested to interrupt the inflammation cascade and stop the progression of the cytokine storm. Inflammatory storms have some specific monitoring indicators, including blood routine, white blood cell and granulocyte ratio, C-reactive protein, and procalcitonin. At present, the inflammatory mediators IL-6 and TNF-α can be used as more prominent indicators to reflect the inflammatory storm. According to the Chinese guidelines for the treatment of COVID-19, our patient is considered a severe patient (oxygen saturation <93% at rest and an oxygenation index <200). Combined with the patient's gradually increasing IL-6 at that time, and considering that the patient was in the early and middle stages of the inflammatory storm, the patient was given hemofiltration and blood perfusion HA330-II in order to remove and adsorb inflammatory mediators. There were evident peak levels of cytokines on the 13th day after admission. We have found that the significant time point of IL-6 reduction was the sixth day of hemofiltration and hemoperfusion. However, the proper time for intervention, based on the levels of cytokines, needs further validation. From the actual observation of this patient, we concluded that hemofiltration combined with hemoperfusion plays an important role in the treatment of COVID-19.

Characteristics similar to this case were found in the published studies [2, 10], for instance, high level of inflammatory cytokines (IL-6, IL-8, and TNF-α), and most severe form of secondary hemophagocytic lymphohistiocytosis. Interestingly, we found that the changes in procalcitonin (PCT) (Table 2) were correlated with cytokines. In addition, it returned to normal range even earlier than the cytokines. This suggests that we can employ PCT as a sensitive marker of injury and prognosis of COVID-19. In addition, recent studies have only used hemoperfusion alone, and there are no reports on hemofiltration combined with hemoperfusion in the treatment of COVID-19. Compared with other studies, we found that hemofiltration combined with hemoperfusion can earlier eliminates the inflammatory and anti-inflammatory factors in the patient's circulation. However, the conclusion needs further verification. These present data may provide some novel clues for the treatment of COVID-19.

## Data Availability Statement

The original contributions presented in the study are included in the article/supplementary material, further inquiries can be directed to the corresponding author.

## Ethics Statement

The studies involving human participants were reviewed and approved by the Medical Ethics Committee of the First Hospital of China Medical University. The patients/participants provided their written informed consent to participate in this study. Written informed consent was obtained from the individual(s) for the publication of any potentially identifiable images or data included in this article.

## Author Contributions

YDu: conception, design, and drafting the manuscript. ZQ, JM, DS, LY, BX, WL, and ZX: revising the manuscript. YDe: conception, design, revising it critically for important intellectual content, and final approval of the version to be published. All authors read and approved the final manuscript and substantially contributed to the case report.

## Conflict of Interest

The authors declare that the research was conducted in the absence of any commercial or financial relationships that could be construed as a potential conflict of interest.
